# Supplementary material for: Mitochondrial Protease Oct1p Regulates Mitochondrial Homeostasis and Influences Pathogenicity through Affecting Hyphal Growth and Biofilm Formation Activities in Candida albicans
Source: J Fungi (Basel). 2024 May 30;10(6):391. doi: 10.3390/jof10060391 (PMC11204688; doi:10.3390/jof10060391)
Supplement: Supplementary file 1 [file jof-10-00391-s001.zip › jof-2990947-supplementary.pdf]

Supplementary materials

Table S1. Strains used in this study.

Strain	Genotype*	Parent	Reference
SN152	*arg4Δ/arg4Δ leu2Δ/leu2Δ his1Δ/his1Δ URA3/ura3Δ ∷imm^{434} IRO1/iro1Δ∷imm^{434}*		Noble [17]
oct1Δ∷HIS1	*OCT1oct1Δ∷C.d.HIS1 arg4Δ/arg4Δ leu2Δ/leu2Δ his1Δ/his1Δ URA3/ura3Δ∷imm^{434} IRO1/iro1Δ∷ imm^{434}*	SN152	This study
oct1Δ/Δ	*oct1Δ∷C.m.LEU2/oct1Δ∷C.d.HIS1 leu2Δ/leu2Δ his1Δ/his1Δ URA3/ura3Δ∷imm^{434} IRO1/iro1Δ∷ imm^{434}*	*oct1Δ∷HIS1*	This study
oct1Δ/Δ:OCT1	*oct1Δ/Δ RPS1/rps1*∷CIp30-Num11 promoter-Oct1	*oct1Δ/Δ*	This study

* *C.m.*，*Candida maltosa; C.d.*，*Candida dubliniensis.*

Table S2. The primers used in this study. All primers were designed by SnapGene Viewer software.

Primer Name	**Sequence (5′-3′)**
Primer-2	ccgctgctaggcgcgccgtgACCAGTGTGATGGATATCTGC
Primer-5	gcagggatgcggccgctgacAGCTCGGATCCACTAGTAACG
OCT1-1	TTTTACCAGTTCAAGTAACGC
OCT1-3	CACGGCGCGCCTAGCAGCGGTATTGTAGCTGTTTTTGGGC
OCT1-4	GTCAGCGGCCGCATCCCTGCACAGGAAAAATGTAATTAGCTTG
OCT1-6	ACATTTCTTTGTTACACGGTT
LEU2 Check Left	AGAATTCCCAACTTTGTCTG
LEU2 Check Right	AAACTTTGAAC CCGGCTGCG
HIS1 Check Left	ATTAGATACGTTGGTGGTTC
HIS1 Check Right	AACACAACTGCACAATCTGG
OCT1 ORF Left	CAACAGAGAATGCAATCCGAA
OCT1 ORF Right	CATCAACACAGAAGGCAAC
OCT1 Check Left	ACAGATCAGAGATAGACGTA
OCT1 Check Right	CCTGAGACTAATTTTCCGAAC
OCT1 ReF	CCATTTACTTTGAAAATGAAAGCGGGTATACCACT
OCT1 ReR	CGGGCCCCCCCTCGACTATAAATTGCTATTCTGACCAAT
Clp30 ReF	TCGAGGGGGGGCCCGGTA
Clp30 ReR	TTTCAAAGTAAATGGTATAGGGA
Re up Check Left	CTCTACCTTTCAAGCCATC
Re up Check Right	CATATGTATACCGGATCGTT
Re down Check Left	ATAATTTATCGGGCACTCGTTG
Re down Check Right	CTGTCCTTCTAGTGTAGCCGTA

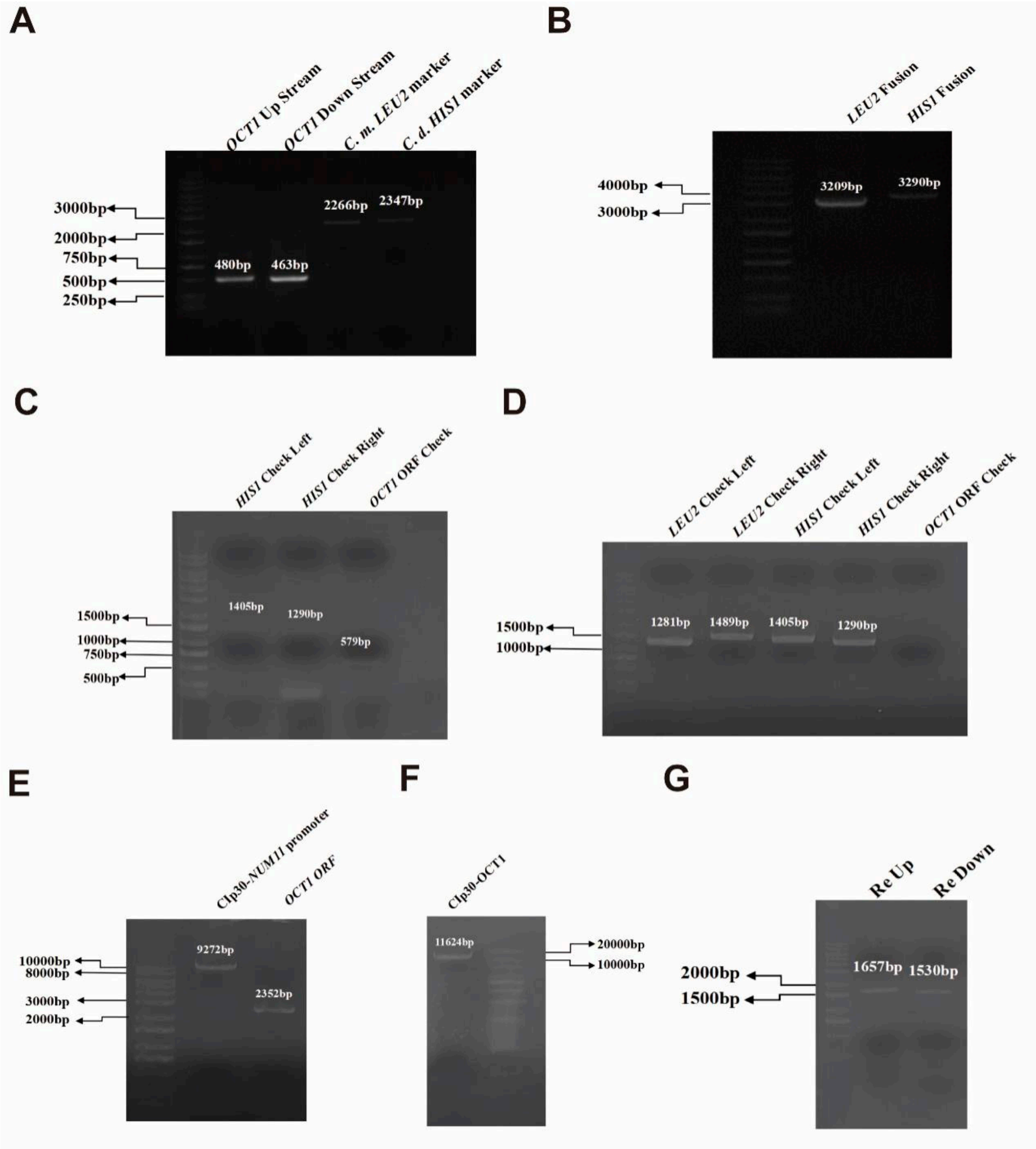

Figure S1. Gel electrophoresis detection plots of the constructed strains (**A**) *OCT1* upstream fragment and downstream fragments as well as the *LEU2* screening marker fragment and the *HIS1* screening marker fragment; (**B**) Fusion products of upstream and downstream *OCT1* fragments and screening markers; (**C**) Identification of the *oct1*Δ∷*HIS1* strain using suite PCR; (**D**) Identification of the oct1Δ/Δ strain using suite PCR; (**E**) CIp30- Num11 promoter fragment and *OCT1* ORF. (**F**) CIp30-Num11 promoter fragment and *OCT1* ORF homologous recombination using *Stu I* single enzyme cleavage product. (**G**) Identification of the *oct1*Δ/Δ:*OCT1* strain.

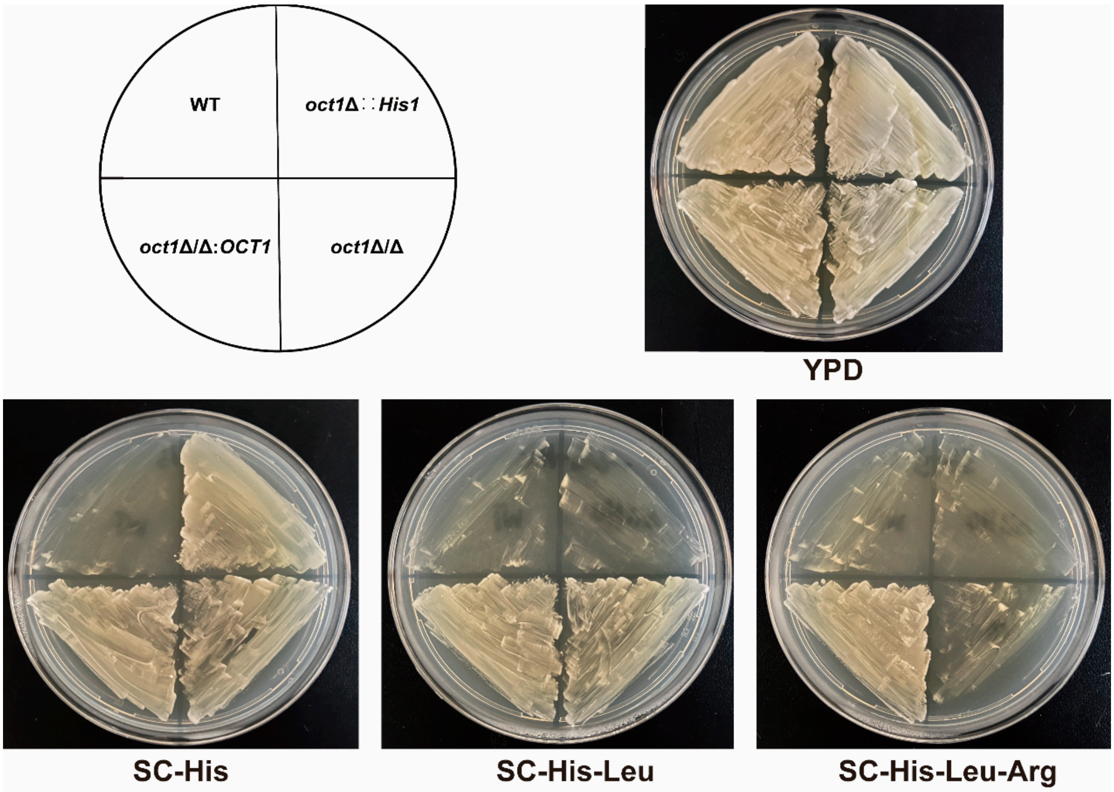

Figure S2. Growth of WT, *oct1*Δ∷*HIS1*, *oct1*Δ/Δ, and *oct1*Δ/Δ:*OCT1* in YPD, SC-His, SC-His-Leu, and SC-His-Leu-Arg medium at 30 °C for 48 h, respectively. The wild type is a nutrition-deficient strain that is unable to synthesize three essential amino acids: leucine, histidine, and arginine. Other strains were constructed through homologous recombination using the synthetic genes of these three amino acids as screening markers. All four strains exhibited normal growth on the glucose-enriched solid medium YPD (Yeast extract peptone). On the SC-His medium that includes leucine and arginine but lacks histidine, the WT could not grow. On the SC-His-Leu medium lacking leucine and histidine, neither the WT nor oct1Δ∷HIS1 could grow. On the SC-His-Leu-Arg medium lacking leucine, histidine, and arginine, only oct1Δ/Δ:OCT1 can grow normally.

Table S3. Plasmids used in this study.

Plasmid	Genotype	Reference
pSN40	*C.m.* with *LEU2* screening marker，Kana^r	Noble [17]
pSN52	*C.d.* with *HIS1* screening marker，Kana^r	Noble [17]
CIp30	the integrating vector for *C. albicans*，Ura3，Amp^r	Murad [10]
CIp30-Num11N	Part of the length *NUM11N* gene in CIp30	This study
CIp30- Num11 promoter - Oct1	*OCT1* gene ORF in CIp30-*NUM11* promoter	This study

Table S4. The primers used in this study. All primers were designed by SnapGene Viewer software.

Gene	Forward primer (5′ to 3′)	Reverse primer (5′ to 3′)
β-actin1-candida albicans	TGCTGAACGTATGCAAAAGG	TGAACAATGGATGGACCAGA

ALS1	TTGGGTTGGTCCTTAGATGG	ATGATTTCAAAGCGTCGTTC
ALS3	CCTATACCACTGCTACTACCGTTA	GTATGGTTGGTGTAATGAGGACG
ECE1	GCCATCATCCACCATGCTCCAG	CAGGAACAGTAGGTGCTTGGTCAG
FTR1	GGTGGTAGTTCCGACCCTCAATTG	GAACGCACCGATGAAAGCACAAC
β-actin1-mouse	CGTAAAGACCTCTATGCCAACA	AGCCACCAATCCACACAGAG
TNF-α	GCATGATCCGAGATGTGGAACTGG	CGCCACGAGCAGGAATGAGAAG
IL-6	CTGCAAGAGACTTCCATCCAG	AGTGGTATAGACAGGTCTGTTGG
IL-10	ACTGGCATGAGGATCAGCAG	AGAAATCGATGACAGCGCCT

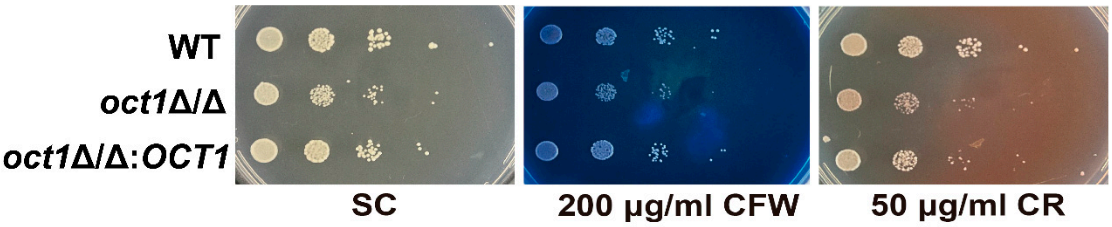

Figure S3. Stress test of different cell wall reagents for each strain.

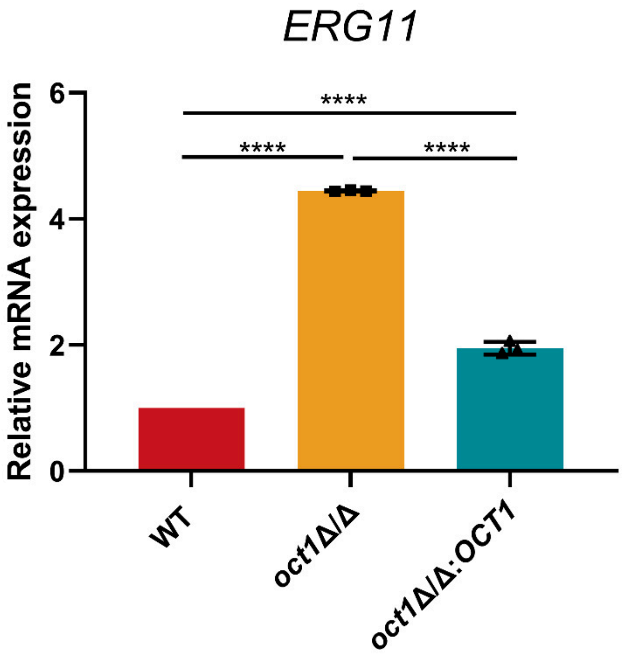

Figure S4. Transcript levels of *ERG11* gene for each strain. （****P < 0.0001）
